# Prognostic Factors Among Patients with Non-metastatic Adrenocortical Carcinoma

**DOI:** 10.5812/ijem-159772

**Published:** 2025-04-30

**Authors:** Haruyuki Ohsugi, Nae Takizawa, Takahiro Nakamoto, Takao Mishima, Katsunori Uchida, Hidefumi Kinoshita

**Affiliations:** 1 Department of Urology and Andrology, Kansai Medical University Hospital, Osaka, Japan; 2 Department of Pathology and Laboratory Medicine, Kansai Medical University, Osaka, Japan; 3 Department of Urology and Andrology, Kansai Medical University Medical Center, Osaka, Japan

**Keywords:** Adjuvant Chemotherapy, Adjuvant Mitotane, Adrenocortical Carcinoma, ENSAT Stage

## Abstract

**Background:**

Adrenocortical carcinoma (ACC) is a very rare and aggressive disease with limited systemic therapeutic options.

**Objectives:**

Treatment with adjuvant mitotane is common after resection of ACC; however, high-risk patients often experience early recurrence. The risk factors for recurrence after surgery were analyzed in patients with non-metastatic ACC.

**Methods:**

We retrospectively reviewed the medical records of 20 patients who were treated for ACC between 1994 and 2023 at Kansai Medical University Hospital or Kansai Medical University Medical Center in Osaka, Japan. We studied the recurrence-free survival (RFS) rates of a subset of 15 patients with non-metastatic ACC [European network for the study of adrenal tumors (ENSATs) stage I-III]. Statistical analyses included the Kaplan-Meier survival analysis and the Cox proportional hazard model.

**Results:**

Of the 15 patients with non-metastatic ACC, nine patients (60%) experienced recurrence. The median RFS was seven months, and all recurrences occurred within 24 months. In all cases, the site of recurrence was the lungs or liver. Univariate analysis showed that ENSAT stage III classification [hazard ratio (HR) 6.974, P = 0.007] was the only factor that made a statistically significant difference to RFS. Although five of the six ENSAT stage III patients took adjuvant mitotane, all experienced recurrence.

**Conclusions:**

In patients with non-metastatic ACC, a diagnosis of ENSAT stage III is the only factor that significantly affects RFS. In these patients, adjuvant mitotane is likely insufficient to prevent recurrence.

## 1. Background

Adrenocortical carcinoma (ACC) is a very rare, aggressive malignant tumor found in the cortex of the adrenal gland, with a high rate of recurrence even after complete resection (1, 2). However, systemic therapeutic options are limited, and complete surgical resection remains the best intervention for the long-term survival of patients with localized ACC (3, 4). Adjuvant mitotane is currently the only treatment used after resection of ACC; however, high-risk patients often experience early recurrence (5).

When recurrence occurs as solitary metastasis or oligometastasis, metastasectomy is a therapeutic option (6, 7). However, when there is a recurrence of multiple metastases or tumors are unresectable, the main treatment is systemic chemotherapy. A randomized study found that a combination of etoposide, doxorubicin, and cisplatin plus oral mitotane (EDP-M) had a positive effect on progression-free survival compared to mitotane plus streptozocin in patients with advanced ACC (8). To improve early systemic intervention with chemotherapy in high-risk patients with non-metastatic ACC, it is necessary to identify prognostic factors associated with recurrence and recognize recurrence patterns.

## 2. Objectives

Recent studies suggest that nomograms or prognostic systems can predict recurrence risk after surgical resection of ACC (9, 10). These studies included a large number of patients and may be useful for classifying the prognosis of patients in clinical settings. However, their assessment of individual recurrence patterns is insufficient. Additionally, there is limited clinical data from Japanese patients with ACC (11, 12). In this study, we analyze detailed recurrence patterns retrospectively and evaluate postoperative recurrence risk in patients with non-metastatic ACC.

## 3. Methods

### 3.1. Patient Selection

The medical records of 20 patients diagnosed and treated for ACC at Kansai Medical University Hospital and Kansai Medical University Medical Center between December 1995 and December 2023 were retrospectively reviewed. The diagnosis of ACC and Weiss criteria were determined based on the 2004 WHO classification of tumors of endocrine organs using hematoxylin- and eosin-stained slides (13). The Ki-67 Index was analyzed by immunohistochemistry. Old pathological specimens prior to 2006 were reviewed by urologic pathologists (T.N. and K.U.) who were blinded to the clinical outcome. The staging system was based on the European network for the study of adrenal tumors (ENSATs) classification system and included four stages: Stage I [presence of a tumor with a diameter of ≤ 5 cm (T1N0)], stage II [presence of a tumor with a diameter of > 5 cm (T2N0)], stage III [positive lymph node status (N1), infiltration into the surrounding tissue (T3), or the presence of tumor invasion into adjacent organs or venous tumor thrombus in the vena cava or renal vein (T4)], and stage IV [presence of distant metastases (M1)] (14). Five patients with stage IV, who had metastasis at diagnosis, were excluded from this study. A total of 15 patients with non-metastatic ACC (ENSAT stages I-III) were analyzed. This study was approved by the institutional review board of Kansai Medical University Hospital, Japan (approval No. 2015607).

### 3.2. Covariates and Outcomes

Clinical data, such as age at the time of surgery and the date of recurrence, were obtained from patient medical records. Demographic data included age, sex, tumor size, preoperative hormonal activity, surgical approach, surgical side, and pathological outcomes [assessed via the Ki-67 Index, Weiss criteria, resected margin, and pathological T (pT) stage]. All patients underwent preoperative imaging, including computed tomography (CT) or magnetic resonance imaging, to evaluate the clinical stage and the presence of distant metastasis. All patients participated in postoperative follow-up appointments for more than one year, with visits every three to four months. Whole-body CT imaging was performed at each visit; if imaging revealed new distant lesions, patients were considered to have recurrent disease.

### 3.3. Statistical Analysis

The primary measure of outcome was recurrence-free survival (RFS), which was defined as the time from surgery to initial recurrence on imaging. All continuous data are shown as median values and interquartile ranges (IQRs). The RFS and overall survival (OS) analyses were assessed using the Kaplan-Meier method’s log-rank test. Multiple comparisons were performed using a Bonferroni correction as a P-value adjustment method. The associations between clinical factors and recurrence were analyzed using a logistic regression model. Hazard ratios (HRs) were estimated with the Cox proportional hazard model and were reported as relative risks with corresponding 95% confidence intervals (CIs). All statistical analyses were performed using EZR software (Saitama Medical Center, Jichi, Japan), with a P-value < 0.05 considered statistically significant (15).

## 4. Results

### 4.1. Patient Characteristics

The final cohort consisted of 15 patients whose baseline clinical characteristics are summarized in Table 1. Five of the patients were female (33.3%) and 10 were male (66.7%); the median age was 54 years, and the median tumor size was 80 mm. The patients were classified into ENSAT stage I (13.3%; n = 2), stage II (46.7%; n = 7), and stage III (40.0%; n = 6). Of the six patients classified as ENSAT stage III, three were considered pT3 and three were considered pT4, and two had a positive resection margin. No other patients with a positive surgical margin were observed. A clinical summary of the information on patients with non-metastatic ACC is provided in Appendix 1 in Supplementary File.

**Table 1.** Baseline Demographics and Clinical Characteristics of Patients ^a^

Variables	Values (N = 15)
**Age (y), median (IQR)**	54 (19 - 77)
**Sex**	
Female	10 (66.7)
Male	5 (33.3)
**Tumor size (mm), median (IQR)**	80 (37 - 180)
**Preoperative hormonal activity**	
Cortisol	5 (33.3)
Androgen	1 (6.7)
Inactive (including unknown)	9 (60.0)
**Ki67 Index (%) **	
≤ 10	4 (26.7)
> 10	7 (46.7)
Not available	4 (26.7)
**Weiss criteria**	
≤ 5	8 (53.3)
> 5	7 (46.7)
**Resected margin**	
Negative	13 (86.7)
Positive	2 (13.3)
**Pathological T stage**	
pT1	2 (13.3)
pT2	7 (46.7)
pT3	3 (20.0)
pT4	3 (20.0)
**ENSAT stage**	
Stage I	2 (13.3)
Stage II	7 (46.7)
Stage III	6 (40.0)
**Surgical approach**	
Laparoscopy	7 (46.7)
Open	8 (53.3)
**Surgical side**	
Right	7 (46.7)
Left	8 (53.3)

Abbreviations: IQR, interquartile range; ENSAT, European network for the study of adrenal tumor; pT, pathological T.

^a^ Values are expressed as No. (%) unless indicated.

### 4.2. Recurrence Patterns in Patients with Non-metastatic Adrenocortical Carcinoma After Surgery

Of the 15 patients with non-metastatic ACC, nine patients (60%) experienced recurrence after surgery. The median RFS time was seven months. There were six cases of lung metastasis, four cases of liver metastasis, and one case of both. Six of the seven stage II patients received adjuvant mitotane treatment; three of these patients experienced recurrence. The stage II patients without recurrence (n = 4) were all treated with adjuvant mitotane. Although five of the six stage III patients received adjuvant mitotane treatment, all six patients experienced recurrence.

### 4.3. Relationship Between Pathological Factors and Recurrence

**Figure 1.**
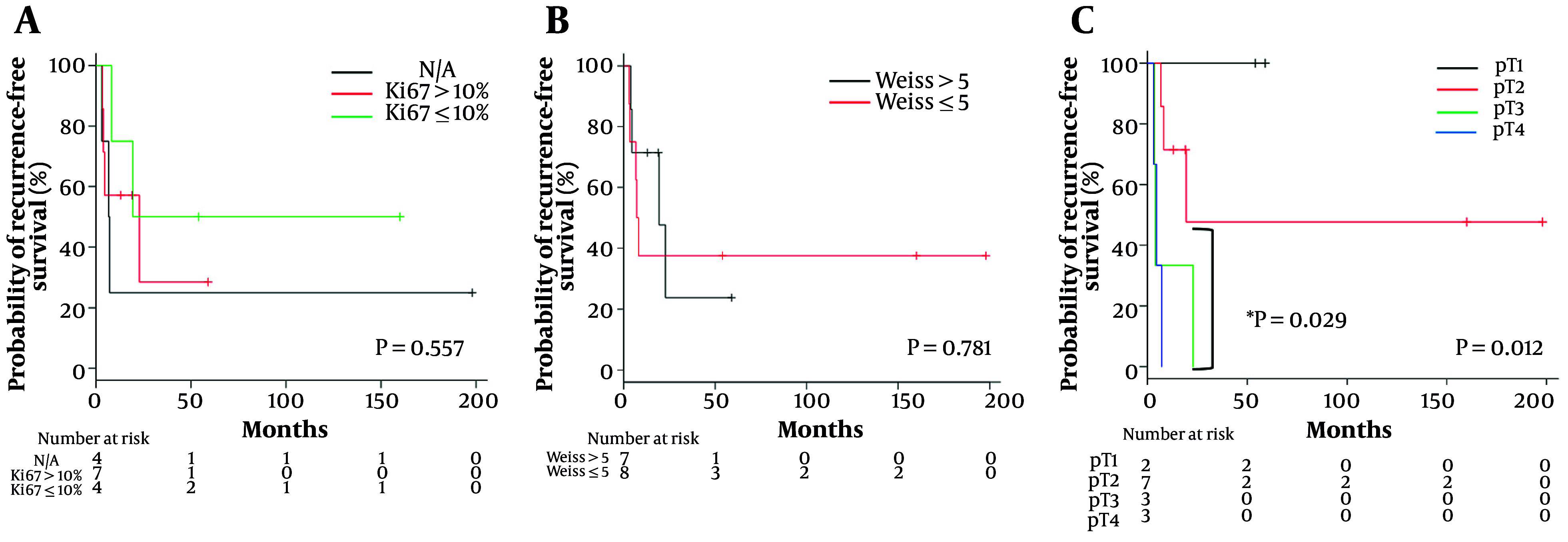
Recurrence-free survival (RFS) in non-metastatic adrenocortical carcinoma (ACC) patients stratified by: A, by Ki-67 Index; B, by Weiss criteria; and C, pathological T (pT) stage (* comparisons between pT2 and pT4).

The Kaplan-Meier survival analysis for RFS was stratified by pathological factors (Figure 1). Although the Ki-67 Index and sum score of the Weiss criteria did not show statistically significant differences (Figure 1A and B), the pT stage was significantly correlated with RFS (P = 0.012), with significant differences between pT2 and pT4 (P = 0.029) (Figure 1C).

### 4.4. Clinical Factors Associated with Recurrence in Patients with Non-metastatic Adrenocortical Carcinoma

**Figure 2.**
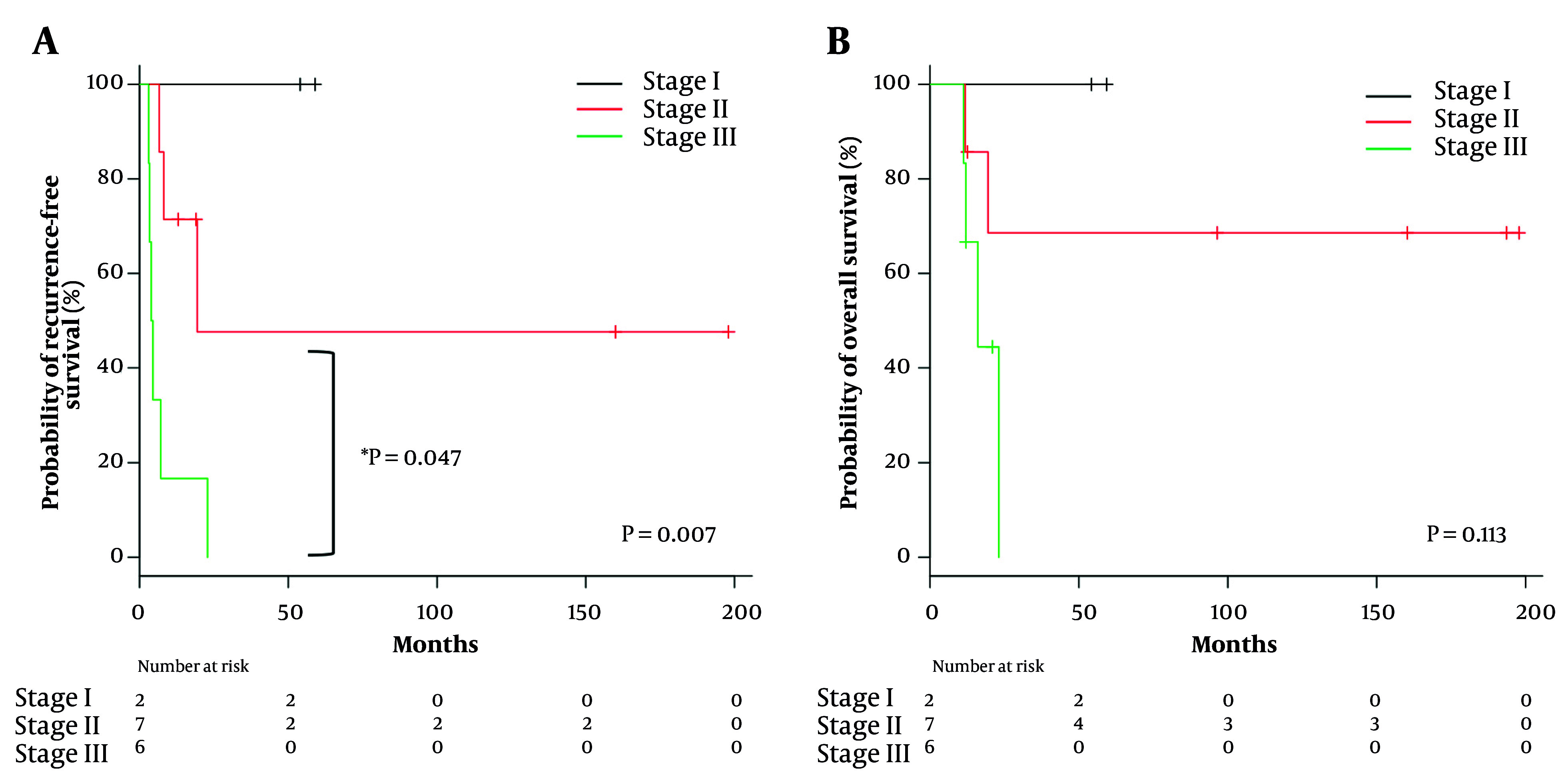
Survival analysis of non-metastatic adrenocortical carcinoma (ACC) patients stratified by European network for the study of adrenal tumor (ENSAT) staging system: A, recurrence-free survival (RFS) and B, overall survival (OS) (* comparisons between stage II and stage III).

Table 2 presents the univariate analysis of clinical factors associated with recurrence. A classification of ENSAT stage III was the only factor that significantly affected recurrence (HR 6.974, 95% CI 1.68 - 28.94; P = 0.007). The RFS rate was significantly worse in ENSAT stage III patients than in ENSAT stage II patients (P = 0.047) (Figure 2A). The OS rate was also stratified by ENSAT stage, although there was no significant difference (Figure 2B). 

**Table 2.** Univariate Analysis of Clinical Factors Associated with Recurrence

Variables	Univariate Analysis	
	HR (95% CI)	P-Value
**Age; y (< 60 vs. ≥ 60)**	0.75 (0.20 - 2.87)	0.678
**Sex (female vs. male) **	0.68 (0.17 - 2.79)	0.595
**Hormonal activity (absent vs. present) **	2.33 (0.62 - 8.81)	0.212
**Tumor size; mm (< 60 vs. ≥ 60)**	2.29 (0.56 - 9.33)	0.246
**ENSAT stage (I-II vs. III)**	6.97 (1.68 - 28.94)	0.007
**Surgical approach (open vs. laparoscopy)**	0.64 (0.17 - 2.42)	0.515
**Surgical side (right vs. left)**	1.01 (0.27 - 3.77)	0.994

Abbreviations: HR, hazard ratio; ENSAT, European network for the study of adrenal tumor.

## 5. Discussion

In the present study, we found that ACC patients classified as ENSAT stage III (pT3 or pT4 without metastasis) had a high risk of recurrence that was not mitigated by adjuvant mitotane treatment. All metastatic sites of recurrence were in the lungs or liver, and recurrence occurred within two years of the initial treatment. Therefore, follow-up imaging for patients with high-risk non-metastatic ACC should focus on these organs from the early postoperative period.

Because ACC is a very rare tumor, there have been few reports summarizing treatment and recurrence outcomes. Clinical data for the Japanese population is especially limited. However, 10 Japanese university hospitals recently reported ACC treatment data for 46 patients (11). This relatively large cohort study showed that the resection of the primary site in stage IV was associated with prolonged survival. However, stage IV was defined using the 7th edition of the American Joint Committee on Cancer (AJCC) staging system, which means that stage IV includes T4N0M0, T3-4N1M0, or Tany Nany M1 patients (13). Therefore, these results should be interpreted with caution because two groups (T4N0M0 and T3-4N1M0) are defined as stage III in the ENSAT staging system (14). Stage III patients in the ENSAT staging system had a poor prognosis in our study, which suggests that they require multidisciplinary treatment, including radical resection.

Surgical resection is a crucial initial treatment for patients with non-metastatic ACC (15). Unfortunately, patients who have received a complete resection often experience postoperative recurrence. Mitotane is the only approved treatment to prolong RFS in patients with radically resected ACC (5). Therefore, mitotane has been administered as a drug to prevent postoperative recurrence for many years. However, the ADIUVO study, which was the first study to use mitotane in a controlled, randomized trial, found that RFS and OS did not differ between the adjuvant mitotane arm and the observational arm (16). One possible reason that treatment with mitotane did not improve patient outcomes is that the patients were considered to be low-grade (negative resected margin, Ki-67 ≤ 10%, and stages I-III). Although these patients were thought to have a relatively good prognosis, 18 of the 91 registered patients relapsed (a recurrence rate of 20%). Moreover, the 18 patients who relapsed were all ENSAT stage II-III. These results implied that patients in these stages still had a high risk of recurrence even with a negative resected margin and Ki-67 ≤ 10%; therefore, adjuvant mitotane treatment alone might be insufficient to prevent recurrence in these patients. Interestingly, the ADIUVO study found that recurrence occurred within two years in 13 of the 18 patients (72.2%), whether they were treated with adjuvant mitotane or not. These results were similar to our study; both highlight the need for intensive follow-ups that include the lungs and liver in the first two years after surgery.

Even after adjuvant mitotane treatment, the recurrence rate is still about 50% (5), so it is important to understand the prognostic factors for postoperative recurrence. Kim et al. reported that, in addition to the ENSAT stage, which was the only factor associated with recurrence in this study, tumor size ≥ 12 cm, positive nodal status, cortisol-secreting tumor, and capsular invasion were prognostic factors associated with RFS (9). Furthermore, Libé et al. revealed that, in addition to the ENSAT stage, age ≥ 50 years, tumor-related symptoms, and the resection status were factors that contributed to OS (10). Strict postoperative follow-up of these high-risk ACC patients is mandatory. In our cohort, recurrence occurred in all ENSAT stage III patients despite the use of adjuvant mitotane treatment in five out of six cases. These results indicate that mitotane is insufficient to prevent recurrence in patients with a high risk of recurrence.

A recent retrospective analysis has shown that adjuvant treatment with platinum-based chemotherapy is associated with prolonged RFS in ACC patients with a high risk of recurrence (ENSAT stages II-III and a median Ki-67 Index of 30%) (17). Therefore, patients with a high risk of recurrence, including ENSAT stage III patients, may find clinical benefits in adjuvant chemotherapy. Unfortunately, prospective evidence for adjuvant chemotherapy in high-risk ACC patients has not been established yet. To clarify this issue, a clinical trial (ADIUVO-2) comparing the outcomes of high-risk ACC patients receiving mitotane alone versus mitotane combined with cisplatin and etoposide is currently ongoing. This prospective randomized trial will determine the role of adjuvant chemotherapy in ACC patients with a high risk of recurrence.

### 5.1. Conclusions

In conclusion, patients with ENSAT stage III ACC had a high risk of recurrence. Since recurrence in these patients could not be prevented with adjuvant mitotane treatment and there is a high risk of metastasis to the lungs and liver within two years, strict postoperative follow-ups, including whole-body CT scans, should be required for patients with non-metastatic ACC.

### 5.2. Limitations

Our study should be interpreted with caution due to several limitations. First, our results were based on data from a retrospective observational study. Second, blood levels of mitotane were not assessed due to the Japanese insurance system. Third, a multivariate analysis was not performed because of the small number of patients. Fourth, a few cases were documented more than 20 years ago. However, the third and fourth limitations are due to the rarity of ACC tumors. The accumulation of real-world data will solve these problems in the future.

ijem-23-2-159772-s001.pdf

## Data Availability

The dataset presented in the study is available on request from the corresponding author during submission or after publication.
