# Supplementary material for: Prognostic Factors Among Patients with Non-metastatic Adrenocortical Carcinoma
Source: Int J Endocrinol Metab. 2025 Apr 30;23(2):e159772. doi: 10.5812/ijem-159772 (PMC12296635; doi:10.5812/ijem-159772)
Supplement: ijem-23-2-159772-s001.pdf [file ijem-23-2-159772-s001.pdf]

Appendix 1. Clinical summary of the information on patients with non-metastatic ACC

Patient	Age (years)	Sex	Side	Hormonal activity	Tumor size	Surgical approach	pT	pN	ENSAT Stage	Resected margin	Ki67 index	Weiss criteria	Adjuvant Mitotane
1	49	Female	Right	inactive	170mm	open	pT4	pN0	III	positive	N/A	3	+
2	60	Male	Right	inactive	130mm	open	pT2	pN0	II	negative	N/A	4	+
3	68	Male	Left	inactive	160mm	open	pT2	pN0	II	negative	N/A	4	+
4	19	Female	Left	cortisol	80mm	open	pT3	pNX	III	negative	12%	4	+
5	77	Male	Right	cortisol	40mm	laparoscopy	pT4	pNX	III	positive	N/A	3	+
6	26	Female	Left	androgen	110mm	open	pT2	pNX	II	negative	3%	4	+
7	48	Female	Left	cortisol	55mm	laparoscopy	pT2	pNX	II	negative	10%	9	+
8	27	Male	Right	inactive	45mm	laparoscopy	pT1	pNX	I	negative	11%	6	+
9	66	Male	Left	inactive	37mm	laparoscopy	pT3	pN1	III	negative	70%	7	-
10	77	Female	Right	cortisol	110mm	open	pT2	pNX	II	negative	10%	5	-
11	47	Female	Left	inactive	38mm	laparoscopy	pT1	pNX	I	negative	10%	4	-
12	77	Female	Left	inactive	180mm	open	pT2	pNX	II	negative	30%	9	+

13	43	Female	Left	cortisol	90mm	laparoscopy	pT3	pNX	III	negative	20%	6	+
14	71	Female	Right	unknown	50mm	laparoscopy	pT2	pNX	II	negative	20%	7	+
15	54	Female	Right	inactive	80mm	open	pT4	pNX	III	negative	40%	9	+
